# Estimating extra length of stay and risk factors of mortality attributable to healthcare-associated infection at a Chinese university hospital: a multi-state model

**DOI:** 10.1186/s12879-019-4474-5

**Published:** 2019-11-20

**Authors:** Qian Zhou, Lili Fan, Xiaoquan Lai, Li Tan, Xinping Zhang

**Affiliations:** 1 School of Medicine and Health Management, Tongji Medical College, Huazhong University of Science and Technology, Wuhan City, Hubei Province People’s Republic of China; 2 Department of Nosocomial Infection, Tongji Hospital, Tongji Medical College, Huazhong University of Science and Technology, Wuhan City, Hubei Province People’s Republic of China

**Keywords:** Length of stay, Multi-state model, Health-care associated infection, Mortality, Developing country

## Abstract

**Background:**

The current evidence of extra length of stay (LOS) attributable to healthcare-associated infection (HCAI) scarcely takes time-dependent bias into consideration. Plus, limited evidences were from developing countries. We aim to estimate the extra LOS and risk factors of mortality attributable to HCAI for inpatients.

**Methods:**

Multi-state model (MSM) was adopted to estimate the extra LOS attributable to HCAI of each type and subgroup. COX regression model was used to examine the risk of mortality.

**Results:**

A total of 51,691 inpatients were included and 1709 (3.31%) among them developed HCAI. Lower respiratory tract infection and Acinetobacter baumannii were the most prevalent HCAI and causative pathogen in surveyed institute. Generally, the expected extra LOS attributable to HCAI was 2.56 days (95% confidence interval: 2.54–2.61). Patients below 65 had extra LOS attributable to HCAI longer about 2 days than those above. The extra LOS attributable to HCAI of male patients was 1.33 days longer than female. Meanwhile, age above 65 years old and HCAI were the risk factors of mortality for inpatients.

**Conclusions:**

HCAI contributes to an increase in extra LOS of inpatients in China. The effect of HCAI on extra LOS is different among subgroups, with the age below 65, male and medicine department more sensitive.

## Background

Healthcare-associated infection (HCAI) is the infection acquired during the hospitalization in a patient which was not present or incubating before admission [1]. HCAI is an important public health issue due to their association with increasing prevalence, mortality and morbidity, extra length of stay (LOS) and excess cost of care. It was claimed that HCAI was the most frequent adverse event threatening the safety of patients worldwide [2]. The prevalence varied between 3.5 and 12% [3], with approximately 2 million HCAI occurring annually in the US [4]. Roberts, et al. [5] even found that HCAI would double mortality and induce increase of health care costs, with hospitals losing $7453–15,155 or even double for each patient due to HCAI, among which half of the excess cost was associated with prolonged hospitalization [5–7].

The accurate estimation of extra LOS attributable to HCAI is crucial for policy decision making, because the extra LOS is the key driver of increasing cost [8]. Most research investigated LOS attributable to HCAI using time-fixed method like group comparison, matching and regression [9]. However, these estimates related to LOS attributable to HCAI own methodological limitations that neglecting time-dependent bias may result in overestimation of the extra LOS [10]. The accurate estimation needs to consider the competing risks of death, increased LOS for time-dependent bias and so on [11]. Manoukian [12] found that multi-state model (MSM) has been recommended as a technique to avoid time-dependent bias, as the model treats the occurrence of HCAI as time-dependent covariate. Thus, the researches using MSM can provide higher quality evidence compared with other researches using time-fixed method [12].

In addition, the estimate evidence of extra LOS attributable to HCAI was limited in developing countries. Especially, there was a paucity of the relevant study controlling for time-dependence in middle-income countries [2]. In this study, we applied multi-state modeling to estimate the extra LOS attributed to HCAI of each type and subgroup so as to provide evidence of high quality in China.

## Methods

### Setting

The study was conducted in a teaching tertiary hospital in central China, which is on the forefront of clinical practice, scientific research and medical education in China.

### Participants and diagnostic criteria

There were 51,691 patients, free of community acquired infection and hospitalized for at least 48 h in 35 clinical departments in 2017. The HCAI diagnostic criteria used in this work were issued by National Health Commission in China 2001 [13]. HCAI refers to infection acquired after 48 h of admission, unless there is a clear incubation period for the infection before 48 h. And HCAI was diagnosed based on laboratory data, image examinations, clinical symptoms and so on, according to the diagnostic criteria. In our study, the patients hospitalized less than 48 h and getting community acquired infection were excluded [14].

### Statistical analysis

We compared the characteristics between patients with HCAI or not, by the Manne-Whitney U-test and Chi-squared test [7]. Mantel-Haenszel methods were also used to calculate unadjusted odds ratios and 95% confidence intervals [14].

**Fig. 1:**
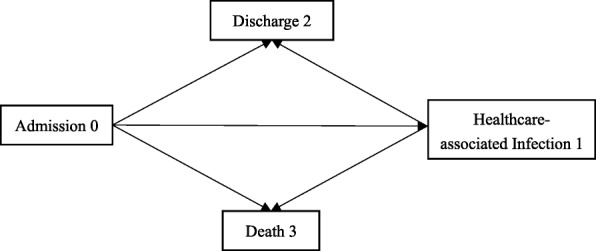
Multi-state model used to determine length of stay

We conducted MSM to estimate extra LOS attributable to HCAI. As LOS attributable to HCAI is mainly caused by the days after HCAI acquired actually, it is necessary to account for the time of HCAI during estimation [11, 15]. Based on the pathway of patients’ states during hospitalization, MSM provides a weighted average of the LOS [16]. In the 4-state model, we treated admission without infection as state 0, HCAI acquisition as state 1, discharge as state 2, and in-hospital death as state 3 (Fig. 1). Transitions between states were determined by time-varying hazards, which were estimated using the Aalene-Johansen estimator [17]. As for patients who got more than one infection during hospitalization, we only estimated the first infection in MSM [15].

We assessed the related individual and departmental factors on the estimates of extra LOS by subgroup [18], including types of HCAI and patient characteristics such as age, gender and department. Those factors were investigated to have significant influence on LOS and the risk of acquiring HCAI [19, 20]. When we estimated the extra LOS attributable to a specific type of HCAI such as bloodstream infection, we excluded surgical site infection, lower respiratory tract infection and other types. As we calculated the extra LOS attributable to HCAI varying patient characteristics such as female, we only included the female patients belonging to the needed scope. Crude extra LOS was calculated by the mean of LOS without HCAI subtracted the mean of LOS with HCAI.

Descriptive data analysis was performed using IBM SPSS Version 20.0 (IBM, New York, NY, USA). MSM analyses were performed using R (Team R Development Core: http://cran.r-project.org/). The variance and confidence intervals in the extra LOS were estimated using 1000 bootstrap resamples.

To examine the risk of reaching those states, we used a COX regression model adjusted for related factors, with LOS as the time variable to avoid time-dependent bias. HCAI was also included in the model as a possible predictor of death or discharge. Schoenfeld residuals were used to test the proportional risk assumption [21].

## Results

### Characteristics of participants and reported causative pathogens on the major types of HCAI

Among 51,691 patients, 1709 cases of HCAI (3.31%) experienced 1914 episodes of infection (1.12 per person). Percentage of each type of HCAI patients among admissions was presented as follows: lower respiratory tract infections (968; 50.57%), bloodstream infection (278; 14.52%), urinary tract infections (239; 12.49%), upper respiratory tract infections (71; 3.71%), surgical site infections (56; 2.93%), skin, soft tissue (48; 2.51%).

Table 1 summarizes the characteristics of patients with and without HCAI and their comparison. Males were significantly more likely to acquire HCAI compared to females (unadjusted OR: 1.1; 95% CI: 1.00–1.23). Patients older than 65 were significantly at risk of HCAI compared to those younger (unadjusted OR: 1.56; 95% CI: 1.40–1.73). Those who had HCAI were also more likely to die in the hospital than those without HCAI (unadjusted OR: 7.94; 95% CI: 6.30–10.00). Meanwhile, HCAI showed varied incidence in different departments. Patients acquired HCAI were prone to be older and have longer LOS compared with those not.

**Table 1:** Characteristics of patients with and without HCAI (n, %)

Characteristic	HCAI (*N* = 1709)	No HCAI (*N* = 49,982)	Total (*N* = 51,691)	*P*-value
Gender				
Female (n, %)	616 (36.04%)	19,271 (38.56%)	19,887 (38.47%)	0.036
Male	1093 (63.96%)	30,711 (61.44%)	31,804 (61.53%)	
Age				
Mean (n, SD)	54.1 (19.95)	50.64 (18.36)	50.76 (18.42)	< 0.001
< 65	1201 (70.28%)	39,299 (78.63%)	40,500 (78.35%)	< 0.001
> =65	508 (29.72%)	10,683 (21.37%)	11,191 (21.65%)	
Department				
Medicine	297 (17.38%)	11,848 (23.70%)	12,145 (23.50%)	< 0.001
Surgical	700 (40.96%)	27,022 (54.06%)	27,722 (53.63%)	
ICU	386 (22.59%)	1808 (3.62%)	2194 (4.24%)	
Pediatric	96 (5.62%)	1471 (2.94%)	1567 (3.03%)	
Psychiatric	157 (9.19%)	6256 (12.52%)	6413 (12.41%)	
Chinese traditional medicine	73 (4.27%)	1577 (3.16%)	1650 (3.19%)	
LOS				
Mean (SD)	28.71 (23.501)	13.90 (12.418)	14.51 (13.201)	< 0.001
Discharge outcome				
Discharge alive	1614 (94.44%)	49,614 (99.26%)	51,228 (99.10%)	< 0.001
Inpatient mortality	95 (5.56%)	368 (0.74%)	463 (0.90%)	

Table 2 represents reported causative pathogens, sorted by the major 4 types of infection. One or more pathogens were reported for 901 infections. No pathogens were reported for the 1013 infections, which were diagnosed by physical symptoms and radiography and so on. Acinetobacter baumannii, *Klebsiella pneumoniae*, *Staphylococcus aureus* were the most prevalent pathogens resulted in 30.72% of all the HCAI. Acinetobacter baumannii, *Klebsiella pneumoniae*, Enterococcus species, *Staphylococcus aureus* caused most lower respiratory tract infection (LRTI), bloodstream infection (BSI), urinary tract infection (UTI), surgical site infection (SSI), respectively.

**Table 2:** Reported causative pathogens on the major types of HCAI (n, %)

Pathogen	All HCAI (*N* = 1914)	LRTI (*N* = 968)	BSI (*N* = 278)	UTI (*N* = 239)	SSI (*N* = 56)
Acinetobacter baumannii	242 (12.64)	180 (18.60)	23 (8.27)	6 (2.51)	3 (5.36)
*Klebsiella pneumoniae*	180 (9.40)	82 (8.47)	51 (18.35)	20 (8.37)	3 (5.36)
*Staphylococcus aureus*	166 (8.67)	92 (9.50)	48 (17.27)	0	10 (17.86)
Canidia Albicans	142 (7.42)	85 (8.78)	9 (3.24)	36 (15.06)	1 (1.79)
Enterococcus species	87 (4.55)	5 (0.52)	21 (7.55)	42 (17.57)	2 (3.57)
*Pseudomonas aeruginosa*	82 (4.28)	54 (5.58)	5 (1.80)	7 (2.93)	1 (1.79)
*Escherichia coli*	65 (3.40)	12 (1.24)	18 (6.47)	27 (11.30)	1 (1.79)
Candida spp.	55 (2.87)	22 (4.34)	6 (2.52)	21 (20.92)	0
C.tropical	52 (2.72)	20 (2.07)	1 (0.36)	29 (12.13)	0
Aspergillus spp.	33 (1.72)	30 (3.10)	1 (0.36)	0	0
Coagulase-negative Staphylococcus spp.	25 (1.31)	6 (0.62)	15 (5.40)	0	1 (1.79)
Neisseria	19 (0.99)	1 (0.10)	0	0	0
*Streptococcus viridans*	17 (0.89)	0	2 (0.72)	0	0
Saccharomyces	17 (0.89)	6 (0.62)	0	10 (4.18)	0
Stenotrophomonas maltophilia	16 (0.84)	12 (1.24)	2 (0.72)	1 (0.42)	0
Enterobacter Hormaeche and Edwards	14 (0.73)	6 (0.62)	5 (1.80)	0	0
Bacillus proteus	8 (0.42)	2 (0.21)	0	3 (1.26)	0
Serratia spp.	8 (0.42)	5 (0.52)	1 (0.36)	0	2 (3.57)
Acinetobacter sp	8 (0.42)	3 (0.31)	1 (0.36)	0	0

*HCAI* healthcare-associated infection, *LRTI* lower respiratory tract infection, *BSI* bloodstream infection, *UTI* urinary tract infection, *SSI* surgical site infection

### Length of stay

LOS mean comparisons and estimates from MSM are shown in Table 3. It indicated that the crude extra LOS attributable to HCAI was 14.72 days. Meanwhile according to MSM, the expected LOS due to HCAI was 2.56 days (95% CI: 2.54–2.61) based on a standard error of 0.42. SSI had the largest impact on the hospitalized patients, whose extra LOS was longer than other sites of HCAI, with extra LOS 14.88 days (95% CI: 14.57–15.19). In contrast, UTI added the less extra LOS among the four types, with extra LOS 0.34 days (95% CI: 0.29–0.42). As the most prevalent HCAI in China, the extra LOS attributable to LRTI was a little higher than average level, with extra LOS 2.66 days (95% CI: 2.65–2.73).

**Table 3:** Estimates of extra LOS attributable to the types of HCAI

	Crude extra LOS ^a^	MSM extra LOS	SE	95% CI
HCAI	14.72	2.56	0.42	(2.54, 2.61)
BSI	17.21	3.92	1.60	(3.90, 4.18)
UTI	13.63	0.34	0.75	(0.29, 0.42)
SSI	32.68	14.88	3.49	(14.57, 15.19)
LRTI	13.09	2.66	0.48	(2.65, 2.73)

*HCAI* healthcare-associated infection, *BSI* bloodstream infection, *UTI* urinary tract infection, *SSI* surgical site infection, *LRTI* lower respiratory tract infection

^a^Defined as the difference in mean LOS between patients with and without HCAI or a specific type of HCAI

Table 4 illustrates the average LOS estimated by patient subgroup. The extra LOS was significantly decreasing with the older age. For patients aged < 65, the extra LOS was 3.07 days (95% CI: 3.07–3.16), while it was 1.10 days (95% CI: 1.03–1.14) for those > 65. When acquired with HCAI, male patients with extra LOS 3.07 days (95% CI: 2.97–3.08) were prone to be hospitalized longer than female patients with extra LOS 1.74 days (95% CI: 1.71–1.84). Variability in estimates of extra LOS was noted among five clinical departments. Among them, patients in surgical department who acquired HCAI were hospitalized longer than those not, with extra LOS 5.44 days (95%CI: 5.43–5.56). Patients in psychiatric department had lowest extra LOS of 1.27 days (95%CI: 1.23, 1.29). Meanwhile, patients acquiring HCAI in pediatric, medicine and Chinese traditional medicine department had 3.80 days (95%CI: 3.77, 3.87), 4.49 days (95%CI: 4.15, 4.54), 2.64 days (95%CI: 2.56, 2.78) extra LOS compared those not, respectively.

**Table 4:** Estimates of extra LOS attributable to HCAI of subgroups

	Crude extra LOS ^a^	MSM extra LOS	SE	95% CI
Patient characteristics				
Age				
< 65 y	14.86	3.07	0.53	(3.06, 3.16)
65+ y	16.00	1.10	0.62	(1.03, 1.14)
Gender				
Female	12.01	1.74	0.77	(1.71, 1.84)
Male	16.22	3.07	0.56	(2.97, 3.08)
Department				
Medicine	16.72	4.49	1.00	(4.15, 4.54)
Surgical	19.34	5.44	0.73	(5.43, 5.56)
Pediatric	11.42	3.80	1.18	(3.77, 3.87)
Psychiatric	6.95	1.27	0.51	(1.23, 1.29)
Chinese traditional medicine	14.78	2.64	1.79	(2.56, 2.78)

^a^Defined as the difference in mean LOS between patients with and without HAIs

### Risk of HCAI, discharge and death

In 2017, 463 patients died in the surveyed institute. 95 (25.52%) were infected. The most type of infected patients who died in hospital were LRTI with 51 (11.02%) death and BSI with 25 (5.40%) death. HCAI were 7.94 (ie, = (95/368)/ (1614/49614)) times more likely to die in the hospital than those without HCAI. Namely, 694% more patients acquired HCAI would die compared to those not (calculated in Characteristics of participants and reported causative pathogens on the major types of HCAI section), when not adjusting time-dependent factors. Patients acquired HCAI were 0.13 (ie, = (1614/49614)/ (95/368)) times less likely to discharge. Table 5 presents results using a Cox regression model. It was turned out that patients above 65 years old were more likely to acquire HCAI (HR: 1.383; 95%CI: 1.246, 1.534). Besides patients above 65 years old were more likely to die (HR: 2.704; 95%CI: 2.250, 3.250). HCAI also significantly was the risk factor of death (HR:2.921; 95%CI: 2.307, 3.698), considering the time-dependent biases.

**Table 5:** Risk of HCAI and death using a Cox regression model

Outcome	Predictor	HR	95% CI	*P*-value
HCAI	Age			
	Below 65 years old	1		
	Above 65 years old	1.383	(1.246, 1.534)	< 0.001
DEAD	Age			
	Below 65 years old	1		
	Above 65 years old	2.704	(2.250, 3.250)	< 0.001
	HCAI			
	Not acquire HCAI	1		
	Acquire HCAI	2.921	(2.307, 3.698)	< 0.001

Schoenfeld residuals

HCAI as outcome: test: Age: *p* = 0.837

DEAD as outcome: Global test: *p* = 0.552; detailed test: Age: *p* = 0.295; HCAI: *p* = 0.707

## Discussion

To our best knowledge, this is the first study to estimate the extra LOS attributable to HCAI applying multi-state model in developing countries as well as the risk of HCAI, discharge and death. Considering the main limitations of MSM model that it is difficult to account for other baseline covariates in analysis, we estimate extra LOS by conducting subgroup analysis to understand extra LOS attributable to HCAI clearly.

The distribution of prevalent HCAI in our study is consistent with the previous prevalence from 52 Chinese hospitals and from a teaching hospital [22, 23], where lower respiratory tract infection was the most prevalent HCAI. We also got the similar results of documented HCAI ratio and mortality compared to previous study [24, 25]. As for causative pathogens, we found that Acinetobacter baumannii was most prevalent. In fact, the percentage of Acinetobacter baumannii is high among pathogens causing HCAI in China compared to other countries [26]. On the contrary, *Clostridium difficile*, frequently reported pathogen in the USA, was not reported in this study. That might be owing to samples’ low submission rate and poor diagnosis in China, which was the same condition in the surveyed institute [27, 28].

The extra LOS attributable to HCAI was 2.56 days in this study. The result is quite similar to the research conducted in Australia using regression model to estimate the extra LOS [29]. Compared to the previous studies conducted by the case-compared method in China, the estimates are shorter [30, 31] . The reason might be overestimation of LOS time-dependent bias in most researches. As for studies using MSM in other developed countries, they found that the extra LOS attributable to HCAI ranging from 0.9 to 11.5 days [6, 18]. Some of them is consistent with our study. Different from previous findings that older patients tended to be hospitalized longer than those younger [23], the extra LOS attributable to HCAI for those aged less than 65 is longer than those above 65 when considered time-dependent bias in our study. Some researchers draw the same conclusion as we did [18, 32]. It might be explained by the less relative impact of HCAI on older patients than those younger [32]. In addition, we found male patients had longer extra LOS attributable to HCAI than female patients. Consistent with the extra LOS estimates in German, male patients were hospitalized longer than female [18]. The reason might be that men were at higher risk of BSI and SSI, which was probably due to skin or other anatomical differences [23]. It was suggested that thicker, coarser hair of male contributed to higher BSI and SSI [33]. Meanwhile, we found that BSI and SSI exactly turned out to prolong LOS longer in our study, which was also approved by Angelis GD [34].

The work reveals implications for healthcare researchers and medical staff. We provided a precise estimate of extra LOS attributable to HCAI based on MSM avoiding time-dependent bias. Besides, the accurate estimates improve awareness of medical staff on prevention of HCAI. And a wide range of infection rates can reduce by preventive measures [35]. Thus, medical staff should pay more attention to implementing infection prevention measures.

There are limitations in our study. Its findings may not generalize to other settings based on one hospital survey in China. While lots of ICU patients in investigated hospital were often treated both in ICU and general units without clear pathway, so we could not specify them associated with LOS. Hence, we didn’t estimate the extra LOS for patients in ICU [6]. Meanwhile, disease severity such as comorbidity and entering ICU or not was not controlled in the model. The community acquired infection might be potentially omitted.

Future studies would explore a model on estimating excess LOS attributable to HCAI on account of more than one HCAI. The effect of infection with documented microbiological result and not on LOS would also be considered.

## Conclusions

HCAI is attributable to an increase in extra LOS of patient hospitalization, considering the time-dependent bias, as well as the variation including patient age, department and type of infections. The results estimated in our study using MSM help improve the accuracy in estimation of burden of HCAI in the future. Older age and HCAI are risk factors of patient mortality. The interventions to prevent infection during patient hospitalization are required.

## Data Availability

The data used in the study was available from the Department of nosocomial department of Tongji Hospital of Huazhong University of Science and Technology. The datasets are available from administrative permissions from the chief of nosocomial department, who is one of our authors.

## Abbreviations

BSI: Bloodstream infection
HCAI: Healthcare-associated infection
LOS: Length of stay
LRTI: Lower respiratory tract infection
SSI: Surgical site infection
UTI: Urinary tract infection
